# O26 Polysaccharides as Key Players in Enteropathogenic *E. coli* Immune Evasion and Vaccine Development

**DOI:** 10.3390/ijms25052878

**Published:** 2024-03-01

**Authors:** Thiago Jordão da Silva Lemos, Herbert Guimarães de Sousa Silva, José Osvaldo Previato, Lucia Mendonça-Previato, Elisangela Oliveira de Freitas, Angela Silva Barbosa, Marcia Regina Franzolin, Luis Fernando dos Santos, Bruna de Sousa Melo, Geovana Ferreira dos Anjos, Renata Hiromi Nakagima Gonçalves, Marta de Oliveira Domingos

**Affiliations:** 1 Laboratório de Bacteriologia, Instituto Butantan, Avenida Vital Brasil, 1500, São Paulo 05503-900, SP, Brazil; thiago_biomed@hotmail.com (T.J.d.S.L.); herbert.silva.esib@esib.butantan.gov.br (H.G.d.S.S.); angela.barbosa@butantan.gov.br (A.S.B.); marcia.franzolin@butantan.gov.br (M.R.F.); bruna.melo.esib@esib.butantan.gov.br (B.d.S.M.); geovana.anjos.esib@esib.butantan.gov.br (G.F.d.A.); renata-hiromi@hotmail.com (R.H.N.G.); 2 Instituto de Biofísica Carlos Chagas Filho, UFRJ, Avenida Carlos Chagas Filho, 373, Cidade Universitária, Rio de Janeiro 21941-902, RJ, Brazil; previato@biof.ufrj.br (J.O.P.); luciamp@biof.ufrj.br (L.M.-P.); elisangelaooreitas@micro.ufrj.br (E.O.d.F.); 3 Centro de Bacteriologia, Núcleo de Doenças Entéricas, Instituto Adolfo Lutz, Avenida Dr. Arnaldo, 355, São Paulo 01246-000, SP, Brazil; luis.santos@ial.sp.gov.br

**Keywords:** *E. coli* O26, vaccine, enteropathogenic *E. coli*, EPEC, enterohemorrhagic *E. coli*, EHEC

## Abstract

Enteropathogenic *Escherichia coli* (EPEC) produce a capsule of polysaccharides identical to those composing the O-antigen polysaccharide of its LPS (lipopolysaccharide) molecules. In light of this, the impact of O26 polysaccharides on the immune evasion mechanisms of capsulated O26 EPEC compared to non-capsulated enterohemorrhagic *Escherichia coli* (EHEC) was investigated. Our findings reveal that there was no significant difference between the levels in EPEC and EHEC of rhamnose (2.8:2.5), a molecule considered to be a PAMP (Pathogen Associated Molecular Patterns). However, the levels of glucose (10:1.69), heptose (3.6:0.89) and N-acetylglucosamine (4.5:2.10), were significantly higher in EPEC than EHEC, respectively. It was also observed that the presence of a capsule in EPEC inhibited the deposition of C3b on the bacterial surface and protected the pathogen against lysis by the complement system. In addition, the presence of a capsule also protected EPEC against phagocytosis by macrophages. However, the immune evasion provided by the capsule was overcome in the presence of anti-O26 polysaccharide antibodies, and additionally, these antibodies were able to inhibit O26 EPEC adhesion to human epithelial cells. Finally, the results indicate that O26 polysaccharides can generate an effective humoral immune response, making them promising antigens for the development of a vaccine against capsulated O26 *E. coli.*

## 1. Introduction

*Escherichia coli* O26 comprises a group of diarrheagenic bacterial strains that exhibit diverse virulence mechanisms. These strains are categorized as enteropathogenic *E. coli* (EPEC) and enterohemorrhagic *E. coli* (EHEC) subgroups [1]. The range of virulence mechanisms exhibited by these pathogens underlies their impact on public health, with EPEC strains contributing to acute diarrhea and infant mortality in developing countries, while globally, EHEC strains are responsible for outbreaks of bloody diarrhea and hemolytic uremic syndrome (HUS) [2,3,4]. These bacteria are also considered to be emerging pathogens due to specific genetic sequences related to virulence factors present in their genomes. For instance, a pathogenicity island (PAI) almost identical to a PAI present in EPEC O15 was identified in the LEE (locus of enterocyte effacement) region of EHEC O26 [5]. Conversely, a conserved high pathogenicity island (HPI) associated with enhanced virulence in EHEC O26 was detected in EPEC O26 strains [6].

The virulence of *E. coli* associated with the serogroup O26 can also be enhanced by a polysaccharide capsule that shares the same composition as the O26 polysaccharide present in their LPS molecule [7,8,9,10]. This type of capsule has the status of an O-antigen capsule and is classified as a Group 4 capsule [7]. 

It is important to note that there are three pathways responsible for the synthesis and assembly of the O antigen: the Wzx/Wzy, ABC transport and synthase pathway [7]. More than ninety percent of all *E. coli* O-antigens, including the O26 polysaccharide, are synthesized via the Wzx/Wzy pathway [7,11]. The O-antigen that comprises the capsule is also synthesized via the Wzx/Wzy pathway. However, the transport of the capsular O-antigen differs from that of the LPS O-antigen [7,11,12,13,14]. For example, in the case of the LPS O-antigen, the O-antigen polymer is ligated to the lipid A-core by WaaL ligase in the outer leaflet of the inner membrane. Subsequently, the mature LPS molecule is translocated to the outer membrane by the Lpt (Lipopolysaccharide transport) machinery [12,13]. In contrast, the O-antigen that composes the capsule is translocated to the outer membrane by proteins encoded by seven genes, *ymcD*, *ymcC*, *ymcB*, *ymcA*, *yccZ*, *etp* and *etk* located in the operon G4C (*gfc*) [14]. 

The O-antigen capsule has never been detected in enteroaggregative *E. coli* (EAEC) and uropathogenic *E. coli* (UPEC) [14]. However, it is present in O78 ExPEC (extraintestinal pathogenic *Escherichia coli*), conferring protection against serum clearance to this pathogen [8]. The O-antigen capsule also provides protection against complement-mediated clearance and facilitates the spreading of *S. sonnei* in the host [10]. Likewise, the O-antigen capsule, along with the LPS O-antigen, protects EPEC against human α-defensin 5 [15]. However, previous studies have shown that humoral immune responses targeting the O-antigen polysaccharides can effectively counteract the protective effects mediated by the capsule [16,17,18]. Thus, in this study, we investigated in EPEC the capacity of the anti-O26 polysaccharide antibodies to neutralize the protection provided by the capsule. Our findings demonstrate the intricate interplay between bacterial capsule and host immunity, and highlight the potential of anti-O26 polysaccharide antibodies as a promising antigen candidate for vaccine formulations against EPEC O26 infections.

## 2. Results

### 2.1. Visualization of the Polysaccharide Capsule

**Figure 1:**
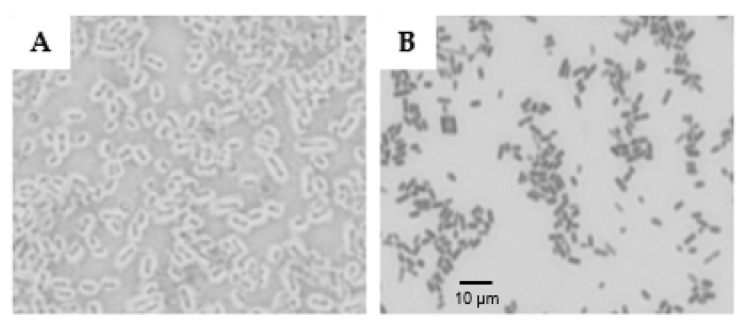
Visualization of bacterial capsule in *E. coli* O26. The presence of a capsule in EPEC O26:H11 (**A**) and EHEC O26:H11 (**B**) was determined by the Maneval’s technique.

The results obtained from Maneval’s staining method demonstrate that the EPEC O26:H11 strain displayed a thick capsule, while the EHEC O26:H11 strain was non-capsulated (Figure 1).

### 2.2. Gas Chromatography Analysis

Gas chromatography analysis of the O26 LPS derived from EPEC and EHEC has shown that the level of rhamnose and N-acetyl-rhamnosamine was similar in both EPEC and EHEC strains. However, the ratio of glucose, heptose and N-acetylglucosamine was found to be higher in EPEC compared to EHEC, with a molar ratio of 10 to 1.69 for glucose (Glc), 3.6 to 0.89 for heptose (Hep), and 4.5 to 2.10 for N-acetylglucosamine (GlcNAc) (Table 1).

### 2.3. Influence of Anti-O26 Polysaccharide Antibodies on E. coli Recognition

**Figure 2:**
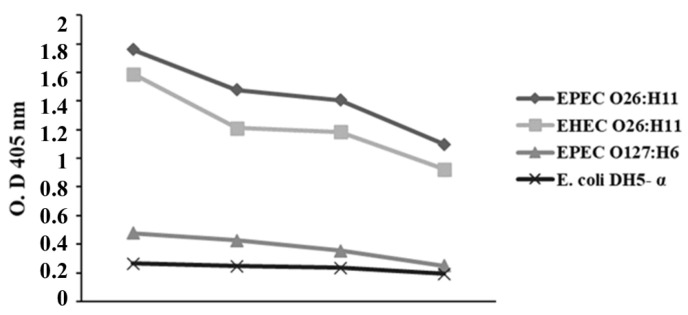
Recognition of *E. coli* strains by anti-O26 polysaccharide antibodies. The ability of anti-O26 antibodies to recognize EPEC O26:H11, EHEC O26:H11, EPEC O27:H6 and *E. coli* DH5α was determined by the ELISA technique.

The capacity of antibodies against O26 polysaccharides to recognize *E. coli* strains was analyzed by ELISA (Enzyme-Linked Immunosorbent Assay). The results demonstrated that antibodies against O26 polysaccharides were able to recognize both EPEC O26:H11 and EHEC O26:H11 strains. However, they did not recognize *E. coli* O127:H6 and *E. coli* DH5-α. (Figure 2).

### 2.4. Influence of Anti-O26 Antibodies on Complement Response

**Figure 3:**
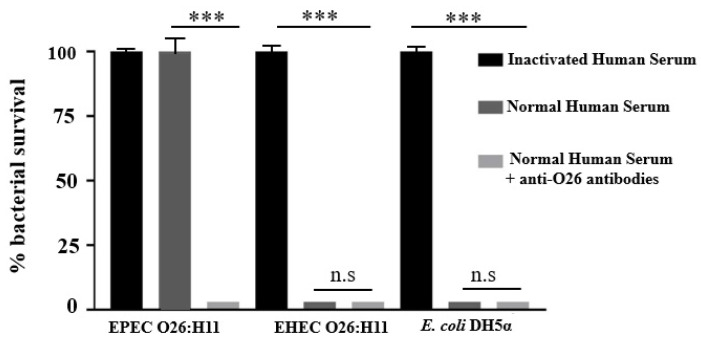
Influence of anti-O26 polysaccharide antibodies on bacterial lysis by complement. Capsulated EPEC O26:H11, non-capsulated EHEC O26:H11, and non-capsulated *E. coli* DH5-α were incubated with inactivated normal human serum, normal human serum (as a complement source), or normal human serum with anti-O26 polysaccharide antibodies. After a 24 h incubation period, bacterial viability was determined by counting the number of colony-forming units (CFUs). Only one sample of each strain was utilized, and the experiment was conducted in quadruplicate. Error bars in the figure represent standard deviation (SD). Unpaired *t*-test: *** (*p* value ≤ 0.001 was considered significant. n.s. = not significant.

The ability of the complement system to lyse capsulated EPEC O26:H11, non-capsulated EHEC O26:H11 and non-capsulated *E. coli* DH5-α was determined by incubating the bacteria with normal human serum as a source of complement. The results demonstrated that non-capsulated O26:H11 EHEC and non-capsulated *E. coli* DH5α were lysed in the presence of normal human serum; however, lysis of the capsulated EPEC O26:H11 strain was observed only when the pathogen was incubated with normal human serum in the presence of anti-O26 polysaccharide antibodies (Figure 3).

### 2.5. Deposition of C3b and C1q on the Bacterial Surface 

**Figure 4:**
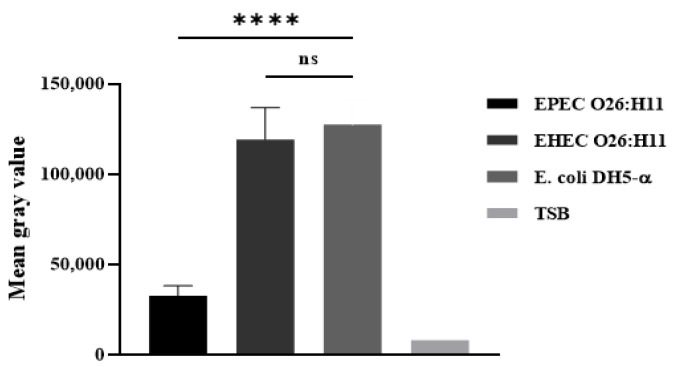
Determination of C3b deposition on *E. coli* surface. The binding of C3b on bacterial surface was determined by the dot blot technique. Nitrocellulose membranes coated with capsulated EPEC O26:H11, noncapsulated EHEC O26:H11 and noncapsulated *E. coli* DH5-α were incubated with normal human serum as a source of complement. Subsequently the membrane was blocked and incubated with goat IgG anti-C3b. After incubation, the membrane was washed and incubated with rabbit anti-goat IgG labelled with peroxidase. The deposition of C3b on the bacterial surface was detected by chemiluminescence (SuperSignalDensitometric) analyses, and the intensity of the signal was determined in pixels by Image J software. The results were plotted as “Mean Gray Values” (average of intensity units in selection). Only one sample of each strain was utilized, and the experiment was conducted in quadruplicate. Error bars in the figure represent standard deviation (SD). Unpaired *t*-test: **** (*p* value ≤ 0.001) was considered significant. n.s. = not significant.

The presence of C3b on capsulated and non-capsulated *E. coli* was determine by the Dot Blot technique after bacterial incubation with normal human serum as a source of complement. The results revealed that the presence of a capsule in EPEC O26 impaired the deposition of C3b on the bacterial surface. Conversely, non-capsulated EHEC O26:H11 and non-capsulated *E. coli* DH5-α strains exhibited C3b deposition on their surfaces (Figure S1 and Figure 4).

**Figure 5:**
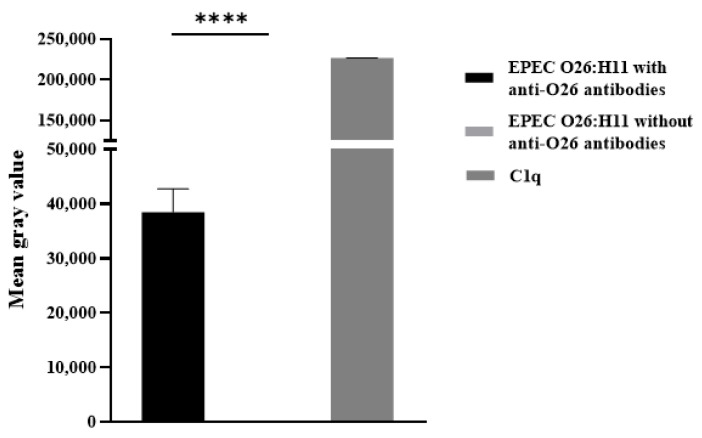
Deposition of C1q on capsulated EPEC O26:H11. The binding of C1q on the bacterial surface was determined by the dot blot technique. Nitrocellulose membrane was coated with capsulated EPEC O26:H11, which was previously incubated for 1 h at 37 °C in the presence or absence of anti O26 polysaccharide antibodies. As a positive control, the membrane was coated with 125 ng of C1q. Subsequently, the membrane was blocked and incubated for 1 h at room temperature with 6.2 µg of C1q in PBS. The membrane was then washed and incubated with goat IgG anti-C1q. After incubation, the membrane was washed and incubated with rabbit anti-goat IgG labeled with peroxidase. The deposition of C1q on the bacterial surface was detected by chemiluminescence (SuperSignalDensitometric) analyses, and the intensity of the signals was determined in pixels by Image J software. The results were plotted as “Mean gray values” (average intensity units in selection). Only one sample of EPEC O26:H11 strain was utilized, and the experiment was conducted in quadruplicate. Error bars in the figure represent standard deviation (SD). Unpaired *t*-test: **** (*p*-value ≤ 0.001) was considered statistically significant.

To investigate the influence of anti-O26 polysaccharide antibodies on complement lysis of capsulated EPEC O26:H11 by the classical pathway, the presence of C1q on antibody-opsonized and non-opsonized EPEC was determined using the Dot Blot technique. The results demonstrated that C1q deposition on the capsulated EPEC O26:H11 surface occurred only when the bacteria were opsonized by anti-O26 polysaccharide antibodies (Figure S2 and Figure 5).

### 2.6. Influence of Anti-O26 Antibodies on Phagocytosis 

**Figure 6:**
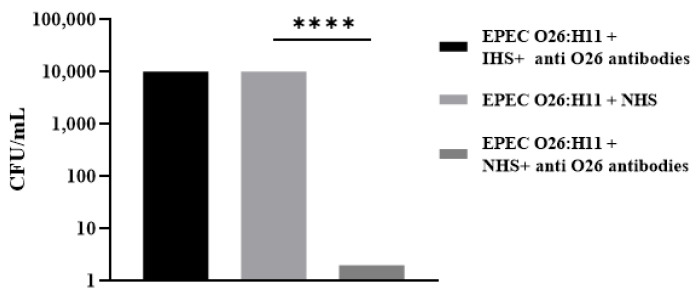
Influence of anti-O26 antibodies on bacterial phagocytosis by macrophages. Macrophages were incubated with capsulated EPEC O26:H11 in the presence or absence of anti-O26 polysaccharide antibodies. Normal human serum (NHS) was used as a source of complement, and inactivated human serum (IHS) was used as a source of inactivated complement. After incubation, the phagocytes were lysed, and the number of ingested bacteria was determined by counting the number of colony-forming units (CFU). Only one sample of EPEC O26:H11 was utilized, and the experiment was conducted in quadruplicate. Error bars in the figure represent standard deviation (SD). Unpaired *t*-test: **** (*p*-value ≤ 0.001) was considered statistically significant.

Considering that the presence of a capsule can impair bacterial recognition by phagocytes [19], the influence of anti-O26 polysaccharide antibodies on the phagocytosis of capsulated EPEC O26:H11 was determined in macrophages J774A.1. The results demonstrated that macrophages were not able to recognize capsulated EPEC O26:H11 in the presence of normal human serum used as a source of complement. However, this inability was overcome when macrophages were incubated with normal human serum in the presence of anti-O26 polysaccharide antibodies (Figure 6). Additionally, even in the absence of normal human serum, the presence of anti-O26 polysaccharide antibodies was enough to help macrophages to recognize EPEC.

### 2.7. Influence of Anti-O26 Antibodies on the Adherence of Capsulated aEPEC O26:H11 to Epithelial Cells

**Figure 7:**
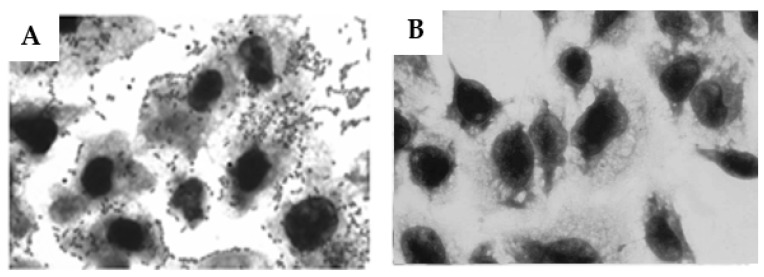
Influence of anti-O26 antibodies on the adherence of capsulated EPEC O26:H11 to epithelial cells. HEp-2 cells were incubated for 3 h with EPEC O26:H11 bacterial culture either alone (**A**), or in the presence of anti-O26 polysaccharide antibodies (**B**). After incubation, the cells were washed and stained for visualization (Ocular ×10, Objective ×100). The original image was magnified 20×.

In the light of the fact that the presence of a capsule can inhibit bacterial adherence [20], we investigated the influence of the anti-O26 polysaccharide antibodies on capsulated EPEC. The results demonstrated that the anti-O26 polysaccharide antibodies were able to inhibit the adherence of capsulated EPEC O26:H11 to the epithelial cells (Figure 7) 

## 3. Discussion

The G4C (*gfc*) operon is present in EPEC and EHEC, indicating that both categories of *E. coli* can produce an O-antigen capsule [14]. However, in this study, an O-antigen capsule was synthesized only by the EPEC O26:H11 strain and not by the EHEC strain. The absence of capsule in the EHEC strain could be associated with several factors, such as gene mutations in the *gfc* operon or inactivation of its promoter [14]. 

Additionally, the expression of the capsule in EHEC and probably EPEC is regulated by Ler, a transcription regulator encoded within the Locus of Enterocyte Effacement (LEE) operon present in both pathogens [9]. 

The presence of the G4C capsule can increase bacterial survival since it can protect the bacteria from phagocytosis and complement lysis [8,10,14]. For example, the O-antigen capsule of EPEC O55 protected the pathogen against macrophage clearance and complement lysis by inhibiting C3b deposition on the bacterial surface [18]. However, doubts have been raised about the O26-antigen capsule’s ability to provide protection to the pathogen, since N-acetylglucosamine (GlcNAc) is part of the capsule’s composition. The doubts are based on the fact that GlcNAc can be recognized by ficolins—a family of pattern recognition receptors (PRRs) associated with MASP (MBL-associated serine protease) [21]. In summary, GlcNAc recognition by ficolin-MASP complex on bacterial surface can lead to complement activation, generation of complement fragments such as C3b and bacterial lysis [21]. 

Our results, however, demonstrated that even though the ratio of GlcNAc was higher in EPEC compared to EHEC, EPEC’s capsule was able to inhibit both deposition of C3b on the bacterial surface and bacterial lysis by the complement system. It is important to note that protection of EPEC by its capsule may be related to its higher ratio of glucose, which can be used by bacteria to decorate GlcNAc in order to evade recognition by the innate immune system [22]. It is also worth noting that the strategy of masking GlcNAc with common monosaccharides is used by several bacteria and can be well illustrated in *L. monocytogenes* serotype 4b (strain M44), where the GlcNAc that covers the wall teichoic acid backbone is decorated with residues of glucose and galactose [22].

In addition, heptose, whose level was also higher in EPEC than EHEC, may be associated with bacterial resistance, since the heptose moieties in the LPS of some bacteria, such as *P. aeruginosa*, are believed to be crucial for bacterial survival, especially in cases of bacteria with heptoseless LPS whose sensitivity to antibiotics and lysis by the complement is increased [23].

Nevertheless, the levels of rhamnose were equivalent in both capsulated EPEC and non-capsulated EHEC. It is worth pointing out that rhamnose is present in bacteria but not in mammals, a fact that categorizes rhamnose as a pathogen-associated molecular pattern (PAMP) and makes rhamnose-associated molecules, potential targets in vaccine formulations [24]. The O26 polysaccharides of EPEC and EHEC also contain RhaNAc, a rare hexose that in *E. coli* has been described only in the side branches of the O3 antigen polysaccharide [11]. 

Despite the observed variations in the ratio of the O26 oligosaccharide units between EPEC and EHEC, the anti-O26 polysaccharide antibodies were able to recognize both pathogens equally. Conversely, it was noted that the anti-O26 polysaccharide antibodies did not recognize an unrelated serogroup or an *E. coli* strain devoid of the O antigen, indicating that these antibodies would preferentially target *E. coli* O26 strains without interfering with unrelated serogroups or commensal *E. coli* strains. 

Finally, anti-O26 polysaccharide antibodies were able to help macrophages recognize capsulated strains, inhibit bacterial adherence to epithelial cells and, as a consequence, disrupt the initial step of bacterial infection. 

Overall, these findings shed light on the complex interactions between O26 *E. coli* strains, their capsules, complement evasion, and the role of anti-O26 antibodies. They provide valuable insights for the development of vaccines targeting the O26 polysaccharide, which could effectively overcome immune evasion mechanisms and enhance protection against O26 *E. coli* infections.

## 4. Materials and Methods

### 4.1. Bacterial Strains

The strains utilized in this study included atypical enteropathogenic *E. coli* (aEPEC) O26:H11 (*eae* positive, Stx negative) isolated in 1988 from a child with diarrhea in São Paulo, SP, Brazil; enterohemorrhagic *E. coli* (EHEC) O26:H11 (*eae* positive, Stx positive), obtained from a human patient with diarrhea in Great Britan, with date of collection unspecified; typical-EPEC O127:H6 (strain E2349/69); and *E.coli* DH5α. All samples were acquired from the *E. coli* collection of the Laboratory of Bacteriology, Instituto Butantan, São Paulo, Brazil. 

### 4.2. Cell Lines

The HEp-2 and J774A.1 cell lines used in this study were obtained from the Instituto Adolfo Lutz, São Paulo, Brazil. The cell lines were previously acquired from the American Type Culture Collection (CCL 2). For maintenance, HEp-2 and J774A.1 cells were grown in DMEM (Dulbecco’s Modified Eagle Medium) and RPMI 1640 (Roswell Park Memorial Institute Medium) respectively. The media were supplemented with 10% calf serum, 1 mM L-glutamine and 50 IU/mL penicillin-streptomycin.

### 4.3. Antibodies against O26 Polysaccharides 

Rabbit serum against O26 polysaccharides was obtained commercially from PROBAC (São Paulo, SP, Brazil).

### 4.4. Pseudo-Capsule Visualization

In order to visualize the capsule, Maneval’s method was employed. Briefly, bacterial cells, scraped from a fully grown agar plate, were transferred into a 10 mL drop of 1% aqueous Congo red solution (Sigma-Aldrich, St. Louis, MO, USA). This suspension was spread across a microscopic glass slide to form a thin film, which was air dried. Then, approximately, 10 mL solution of Maneval [3.33% fenol, 4.44% glacial acetic acid, 2.67% ferric chloride, 0.02% acid fuchsin (Sigma-Aldrich)] was distributed across the slide. After 2 min of incubation, the stain was discarded, the slides air-dried and the capsules were visualized in light microscopy (eyepiece, ×10; objective, ×100). Because the Maneval stain is a negative-stain method, the capsules appear as a transparent halo around the bacteria.

### 4.5. Extraction and Purification of LPS

LPS (20% of bacterial mass) derived either from O26:H11 EHEC or O26:H11 EPEC was extracted using phenol-EDTA-TEA buffer (0.25 M EDTA) and 5% phenol adjusted to pH 6.9 with triethylamine as described by [25]. The cell suspensions were incubated at 37 °C with constant agitation for 1 h and subsequently centrifuged at 10,000× *g* for 1 h. After centrifugation, the supernatants were collected and dialyzed in dialysis tubes with a pore size of 2000-molecular-weight (MW) against running water for 3 days and deionized water for 1 day. After dialysis, the samples were concentrated using a rotary evaporator, clarified by centrifugation at 5000× *g* for 20 min, and lyophilized. For purification, the LPS extracts were solubilized in distilled water and ultracentrifuged at 105,000× *g* for 16 h at 4 °C. The pellets containing purified LPS were solubilized in distilled water and lyophilized. Contaminating nucleic acids were removed by treatment with nucleases (RNase and DNase, Sigma). 

### 4.6. Sugar Analysis

Monosaccharides from LPS (500 µg) were analyzed as their trimethylsilyl (TMS) methyl-glycosides after methanolysis with 0.5 M HCl in methanol at 80 °C for 18 h. The methanolized products were extracted with hexane. The methanolic phase was neutralized by addition of silver carbonate, re-N-acetylated with acetic anhydride (overnight at room temperature in dark), dried under a stream of nitrogen, and trimethylsilylated by addition of bis(trimethylsilyl)-trifluoracetamide (BSTFA)/pyridine (ratio, 1:1) at room temperature for 1 h. The TMS methyl-glycosides were identified by gas-liquid chromatography (GC) and GC-mass spectrometry (GC-MS) on a DB-1 fused silica column (30 m × 0.25 mm/internal diameter) using a temperature program with initial temperature of 120 °C gradually increasing to 240 °C at a rate of 2 °C/min [26,27]. The TMS methyl-glycosides were initially characterized by comparison of their retention times to those of authentic standards and confirmed by GC-MS analysis on a Shimadzu GC 17 A gas chromatograph interfaced with a GC-MS-QP5050 quadruple mass spectrometer.

### 4.7. Serum Resistance Assay

In order to determine the ability of the complement system to lyse the bacterial samples, 5 µL of EPEC O26:H11 and EHEC O26:H11 (1 × 10^13^ CFU/mL) were added to the wells of a 96-well culture plate. The plate was then incubated for 16 h at 37 °C with 15 µL of heat-inactivated serum or 15 µL of normal human serum (Complement Technology, Tyler, TX, USA) in the presence or absence of 50 µL of anti-O26 polysaccharide antibodies. Subsequently, the bacterial viability was determined by counting the number of colony-forming units (CFU) according to the methodology described by Baron and coworkers and the methodology outlined by Beck et al. [28,29]. Normal human serum was used as a source of complement.

### 4.8. C3b Deposition on the Bacterial Surface

The deposition of C3b on the surface of EPEC O26:H11 and EHEC O26:H11 was assessed using the dot blot technique. Briefly, nitrocellulose membranes (0.42 µM) were coated with 2 µL of bacterial samples (1 × 10^13^ CFU/mL). Subsequently, the membranes were blocked for 18 h at room temperature with a solution of 3% BSA in PBS. Subsequently, the membranes were washed three times with washing buffer (PBS containing 0.05% Tween 20) and then incubated for 1 h at 37 °C with 10% normal human serum in incubation buffer (1% BSA in PBS). Following that, the membranes were washed and incubated for 1 h with goat anti-C3b antibodies (Complement Technology, Tyler, TX, USA) diluted 1:5000 in incubation buffer. After incubation, the membranes were washed again and incubated for 1 h with rabbit anti-goat IgG peroxidase conjugate (Sigma-Aldrich) diluted 1:10,000 in incubation buffer. The membranes were then washed, and the deposition of C3b on the bacterial surface was detected by chemiluminescence using SuperSignal West Pico Enhanced Chemiluminescent Substrate (Pierce Biotechnology, Inc.—Thermo Fisher Scientific, Waltham, MA, USA). The intensity of the signals was determined in pixels using Image J software Version 1.5, developed at the National Institutes of Health and the Laboratory for Optical and Computational Institutes (LOCI, University of Wisconsin, Madison, WI, USA), and the results were presented as ‘Mean gray values’ (average intensity units in pixels).

### 4.9. C1q Deposition on the Bacterial Surface

The deposition of C1q on the surface of EPEC O26:H11 was also assessed using the dot blot technique. Nitrocellulose membranes (0.42 µM) were coated with 2 µL of EPEC O26:H11 culture (1 × 10^13^ CFU/mL) previously incubated for 1 h at 37 °C in the presence or absence of anti-O26 polysaccharide antibodies diluted 1/10 in PBS. As a positive control, 2 µL of C1q (125 ng/µL) were directly coated onto the membrane. 

The membrane was blocked and washed as described above, and subsequently, incubated for 1 h with goat IgG anti-C1q (Complement Technology, Tyler, TX, USA) diluted 1:5000 in incubation buffer. After washing, the membranes were incubated for 1 h at room temperature with rabbit anti-goat IgG conjugated with peroxidase (Sigma-Aldrich) diluted 1:10,000 in incubation buffer. Following another round of washing, the deposition of C1q on the bacterial surface was detected by chemiluminescence using SuperSignal West Pico Enhanced Chemiluminescent Substrate (Pierce Biotechnology, Inc.—Thermo Fisher Scientific, Waltham, MA, USA). The intensity of the signals was determined in pixels using Image J software, Version 1.5, developed at the National Institutes of Health and the Laboratory for Optical and Computational Institutes (LOCI, University of Wisconsin, Madison, WI, USA), and the results were presented as ‘Mean gray values’ (average intensity units in pixels).

### 4.10. Phagocytosis

J774A.1 macrophages were seeded in 24-well cell culture plates at a concentration of 10^5^ cells/mL in DMEM supplemented with 10% fetal bovine serum (1 mL/well). The plates were then incubated for 48 h at 37 °C in a 5% CO_2_ incubator. In parallel, bacterial samples (40 µL) containing 10^7^ cells/mL were incubated for 1 h at 37 °C with anti-O26 polysaccharide antibodies diluted 1/10 in 1 mL of DMEM without antibiotics, supplemented with either 10% normal human serum (NHS) or inactivated human serum (HIS). After incubation, the samples were added in triplicate to the macrophage cells and further incubated for 3 h at 37 °C in a 5% CO_2_ incubator. Following incubation, the cells were washed 6 times with sterile PBS and then treated with 50 µg/mL of gentamicin for 30 min. Subsequently, the plates were washed 6 times with sterile PBS, and the macrophages were lysed by incubating the cells with Triton X-100 (Merck) diluted 1/10 in PBS (500 µL/well) for 10 min at room temperature. After incubation, 500 µL of PBS were added to each well to resuspend the lysate. Next, 100 µL of each lysate were serially diluted 10-fold in saline, starting with a dilution of 1 in 10. Ten microliters of each dilution were then plated in triplicate on LB (Luria Broth) agar plates. The plates were incubated overnight at 37 °C, and the number of colony-forming units (CFUs) was determined [28,29].

### 4.11. Inhibition of Bacterial Adhesion to Epithelial Cells

HEp-2 cells were grown to 70% confluence on circular coverslips in wells of 24-well tissue culture plates in the presence of DMEM without antibiotics. Forty microliters of bacterial culture (EPEC O26:H11) at a concentration of 10^7^/mL previously incubated for 1 h at 37 °C with anti-O26 polysaccharide antibodies diluted 1/10 in DMEM containing 2% fetal bovine serum were added in triplicate to the wells (1 mL/well) and incubated for 3 h at 37 °C in 5% CO_2_. As a positive control for bacterial adhesion, the cells were incubated only with bacteria in the absence of antibodies. After incubation, the monolayers were washed 6 times with sterile PBS and then fixed with 100% methanol for 10 min, stained for 5 min with May–Grunwald stain diluted 1:2 in Sorensen buffer, and finally stained for 20 min with Giemsa stain diluted 1:3 in Sorensen buffer. The excess stain was discarded, and the coverslips with the stained cells were air-dried and then affixed to microscope slides for visualization by light microscopy (eyepiece, ×10; objective, ×100).

### 4.12. Statistical Analysis

Statistical analysis was conducted using GraphPad Prism version 8.0.2, GraphPad Software, San Diego, CA, USA) with the unpaired *t*-test. A *p*-value ≤ 0.001 (****) was considered statistically significant. 

## 5. Conclusions

These findings strongly support the notion that O26 polysaccharides are promising antigen targets for developing a vaccine against capsulated *E. coli* O26 strains. By specifically targeting their O26 polysaccharides, such a vaccine has the potential to elicit a humoral immune response capable of neutralizing immune evasion, promoting bacterial clearance, and preventing initial adhesion, thereby offering protection against infections caused by these pathogenic strains

## Figures and Tables

**Table 1:** Monosaccharide composition of O26:H11 EPEC and O26:H11 EHEC LPS.

Monosaccharide	Molar Ratio ^a^	
	EPEC O26:H11	EHEC O26:H11
Rha	2.8	2.5
RhaNAc	1.0	1.0
Glc	10.0	1.69
Hep	3.6	0.89
Kdo	2.6	0.46
GlcNAx	4.5	2.10

^a^ Molar ratio calculated relative to RhaNAC = 1.0; Rha (Rhamnose); RhaNAC (N-acetylrhaminosamine); Glc (Glucose); Hep (Heptose); Kdo (2-keto-3-deoxyoctonate); GlcNAc (N-acetylglucosamine)

## Data Availability

The data presented in this study are available on request from the corresponding author. The data are not available in the repository of the Butantan Institute (https://repositorio.butantan.gov.br (accessed on 30 November 2023).

## Supplementary Materials

The following supporting information can be downloaded at: https://www.mdpi.com/article/10.3390/ijms25052878/s1.
