# Supplementary material for: O26 Polysaccharides as Key Players in Enteropathogenic E. coli Immune Evasion and Vaccine Development
Source: Int J Mol Sci. 2024 Mar 1;25(5):2878. doi: 10.3390/ijms25052878 (PMC10932389; doi:10.3390/ijms25052878)
Supplement: Supplementary file 1 [file ijms-25-02878-s001.zip › ijms-2798257-supplementary.pdf]

Supplementary Materials

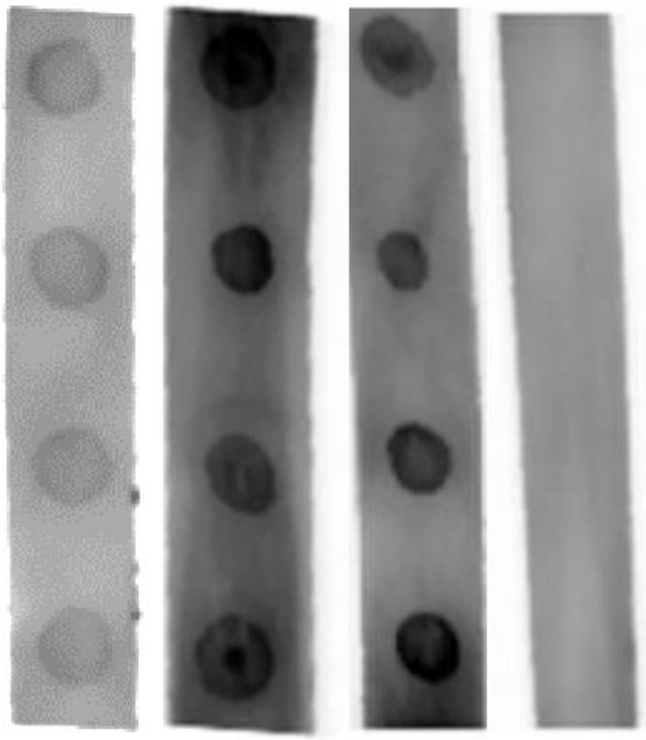

Figure S1. Determination of C3b deposition on *E. coli* surface. The binding of C3b on bacterial surface was determined by the dot blot technique. Nitrocellulose membranes coated with capsulated EPEC O26:H11, non-capsulated EHEC O26:H11 and non-capsulated *E. coli* DH5-α were incubated with normal human serum as a source of complement. Subsequently the membrane was blocked and incubated with goat IgG anti-C3b. After incubation, the membrane was washed and incubated with rabbit anti-goat IgG labelled with peroxidase. The deposition of C3b on the bacterial surface was detected by chemiluminescence (SuperSignalDensitometric) analyses, and the intensity of the signal was determined in pixels by Image J software. The results were plotted as "Mean gray values" (average of intensity units in selection). Only one sample of each strain was utilized, and the experiment was conducted in quadruplicate. Data related to Figure 4 of the manuscript.

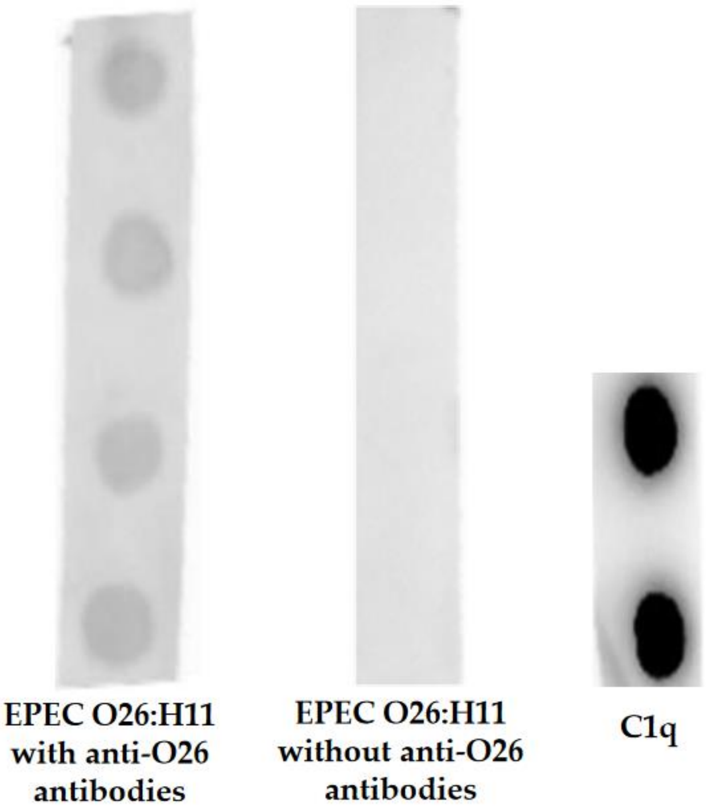

Figure S2. Deposition of C1q on capsulated EPEC O26:H11. The binding of C1q on the bacterial surface was determined by the dot blot technique. Nitrocellulose membrane was coated with capsulated EPEC O26:H11, which was previously incubated for 1 hour at 37 °C in the presence or absence of anti O26 polysaccharide antibodies. As a positive control, the membrane was coated with 125 ng of C1q. Subsequently, the membrane was blocked and incubated for 1 h at room temperature with 6.2 µg of C1q in PBS. The membrane was then washed and incubated with goat IgG anti-C1q. After incubation, the membrane was washed and incubated with rabbit anti-goat IgG labeled with peroxidase. The deposition of C1q on the bacterial surface was detected by chemiluminescence (SuperSignalDensitometric) analyses, and the intensity of the signals was determined in pixels by Image J software. The results were plotted as "Mean Gray Values" (average intensity units in selection). Only one sample of EPEC O26:H11 strain was utilized, and the experiment was conducted in quadruplicate. Unpaired t-test: *** (p-value ≤ 0.05) was considered statistically significant. Data related to Figure 5 of the manuscript.
